# Strategy required: “Maintenance of certification for European radiologists”

**DOI:** 10.1186/s13244-020-00953-9

**Published:** 2021-02-11

**Authors:** Apostolos H. Karantanas, Mario Maas

**Affiliations:** 1 Department of Radiology, University of Crete and Department of Medical Imaging, University Hospital, Heraklion, Greece; 2 Computational BioMedicine Laboratory, Institute of Computer Science, Foundation for Research and Technology-Hellas (FORTH), Heraklion, Greece; 3 Department of Radiology and Nuclear Medicine, Amsterdam University Medical Centres, Univeristy of Amsterdam, Amsterdam, The Netherlands; 4 Amsterdam Movement Sciences Research Institute, Amsterdam, The Netherlands

**Keywords:** Licensure, Education, Continuing, Certification, Radiology

## Abstract

Maintenance of certification (MOC) is thought to be an important tool in assessing and controlling quality of medical professionals. Considerations on the heterogeneity of implementation throughout various National Radiology Associations are described. Testocracy is warned for. The urge for defining strategical steps from a central institute is discussed.

Harmonization of education and training is among the highest priorities of the *European Society of Radiology* (ESR). In this respect, efforts were made in the past that could be beneficial for planning harmonization [1, 2]. Such survey was presented by one of the authors (AHK, *Current status from a survey. Harmonization of training programs in Europe: Myth or reality? European Congress of Radiology, Vienna 2005*), following an initiative of the *European Association of Radiology* (the predecessor of ESR). The results of the study show that there exists a significant heterogeneity among countries with regard to training in radiology, final exams, sub-specialization, and maintenance of certification (MOC). The heterogeneity observed is an important issue not only for assuring the best clinical practice but also for establishing a minimal requisite standard of professional skill as freedom of movement and residence for persons in the EU is the cornerstone of Union citizenship, established by the Treaty of Maastricht in 1992.

Well-being, or good health status, is directly related to the public demand that physicians are fully aware of their need for lifelong learning [3]. The issue of MOC in radiology has been a matter of extensive discussion throughout the world. Most of the reports are from the United States of America (USA), one of the countries most committed to setting high standards in clinical radiology and practice for the benefit of the patients [3–6]. In the USA, an Advisory Board for few subspecialties which did not include Radiology was established as early as 1933 [2]. Few decades later, in 1970, the board transformed to the American Board of Medical Specialties, including Radiology, with a mission, among others, to certify the maintenance of quality of its diplomats [2].

The article by Robert Kwee and Thomas Kwee published in this issue of Insights explores whether the continuing medical education (CME) requirements for radiologists are similar across Europe [7]. They contacted 46 European countries and achieved a response rate of 80%. In the majority of the responses (59%), the radiologist's license is valid only when certain CME points are required, 40 annually, on average. The median license period was 5 years. Eight countries require additional education such as clinical practice, participation in meetings providing quality of practice and clinical audit, and specific courses on radiation safety. The authors conclude that a considerable heterogeneity exists across Europe in regard of the MOC for radiologists.

The authors of this questionnaire study should be congratulated on this investigation, which shows that there are national societies who have not invested on the MOC. The findings of the study give raise to multiple considerations:

1. Countries with a solid tradition in Radiology and large radiological societies have not responded at all.
2. The next step after collecting the data needs to be focusing on how to transform the CMEs onto quality of continuous education and translation to proper professional skills with specific practice performance.
3. Practice of Radiologists in order to achieve a well defined MOC can be performed in designated Educational Centers, i.e., University/Teaching Hospitals, and the duration of license has to be linked to CMEs acquisition and to the performed practice.
4. The ESR and Subspecialty Societies may initiate an effort toward harmonization in agreement with the national societies. The *European Training Curriculum for Radiology*, published by the ESR, has shown a way of creatively acting as members of a big family. The voluntary or obligatory application of the MOC should be discussed. In case of obligatory MOC, failure to meet them should clearly imply well-defined consequences.
5. Validation and standardization of the MOC programs and their impact on improving patient care and adaptation of ongoing medical imaging innovations have not been universally established.
6. There is a variety of ways in which CME credits can be provided to the applicant. Generally, Kirkpatrick’s four-level evaluation model is used as a way of evaluating learners’ outcome in training programs [8]. Let us take a closer look at this model and put it into our own learning experiences. There are four hierarchical levels: (I) the satisfaction of the learner and reactions to the program or teachers. This is the way our largest Radiology Conferences (RSNA, ECR) test participants quality and provide CME credits. (II) measures of knowledge gained, measures of learning by the participant. This is regularly used by journals providing CME credits, where questions are to be answered correctly using an online platform, both international (Radiology, RSNA.org), as national in The Netherlands (IMAGO, imago-nascholing.nl). However, the purpose of CME is behavioral change, since we know that learning programs are founded to make a change in professional practical routine: the concept of life-long learning. (III) assesses the changes in the behavior of the student in relation to the context in which the training took place. (IV) the program’s final result in a much larger context, such as national or international societal impact of the program. We are unaware of the use of Kirkpatrick’s level III or IV in any national or international Radiological community.

In view of these considerations, there is work to be done: it is not only a matter of movement of radiologists across Europe, seeking for a job. It is a matter of Radiology and patient care. Radiology has to keep the leading role in the era of molecular imaging, artificial intelligence, and Precision Medicine. Incorporation of ongoing research, new tools of extracting data, methods of analyzing massive data, and clinical relevance require a continuous effort and sense of duty. We know that radiologists who are not obliged to take part in MOC do so less frequently, which is true in higher percentage for non-academic radiologists [9].

However, we do not seek Testocracy [10]! We know that gaining knowledge is only one of many competences radiologists need to preserve, maintain, or develop. Communications skills or interprofessional behavioral skills are not measured in this way at all [8].

In the end, we are all loyal to Hippocrates' oath: *May I always act so as to preserve the finest traditions of my calling and may I long experience the joy of healing those who seek my help* (11).
